# Assessment of right ventricular systolic function using tricuspid annular-plane systolic excursion in Nigerians with systemic hypertension

**DOI:** 10.5830/CVJA-2010-031

**Published:** 2010-08

**Authors:** KM Karaye, AG Habib, S Mohammed, M Rabiu, MN Shehu

**Affiliations:** Department of Medicine, Bayero University, Kano, Nigeria; Department of Medicine, Aminu Kano Teaching Hospital, Kano, Nigeria; Department of Medicine, Bayero University, Kano, Nigeria; Department of Medicine, Aminu Kano Teaching Hospital, Kano, Nigeria; Department of Medicine, Aminu Kano Teaching Hospital, Kano, Nigeria; Department of Medicine, Aminu Kano Teaching Hospital, Kano, Nigeria; Department of Medicine, Aminu Kano Teaching Hospital, Kano, Nigeria

**Keywords:** right ventricular systolic function, hypertension, TAPSE

## Abstract

**Aim:**

Right ventricular (RV) systolic function in patients with hypertensive heart disease (HHD) is not well characterised. The primary aim of this study was to assess the systolic function of the right ventricle in patients with HHD using tricuspid annular-plane systolic excursion (TAPSE).

**Methods:**

The study was cross-sectional in design and carried out in Kano, Nigeria. Patients were recruited if they had HHD on echocardiography and were at least 15 years of age. Patients with other cardiac pathologies such as ischaemic and valvular heart diseases were excluded. Patients were considered to have abnormal RV systolic function if they had reduced values of TAPSE (< 15 mm). A *p*-value of < 0.05 was considered statistically significant.

**Results:**

A total of 186 patients were serially recruited over seven months. Of these, 131 (70.4%) had normal RV systolic function (group 1) and 55 patients (29.6%) had abnormal function (group 2). Group 2 patients were older (*p* = 0.002) and had a higher prevalence of peripheral oedema (*p* = 0.002), moderate to severe dyspnoea, higher heart rate and lower left ventricular ejection fraction (*p* < 0.001). Atrial arrhythmias were also more prevalent among group 2 patients (*p* < 0.05). The best correlate to TAPSE was the septal mitral annularplane systolic excursion (*r* = +0.541, *p* < 0.001). Several variables such as age predicted the presence of reduced TAPSE.

**Conclusion:**

The study found that almost one-third of patients with HHD in Kano had RV systolic dysfunction as defined by reduced TAPSE, and these patients had a greater prevalence of factors associated with morbidity and mortality.

## Summary

The right ventricle has a complex three-dimensional structure with a crescent-shaped cavity when viewed in cross section. It consists of three parts: the inflow tract, the apical ‘trabecular’ portion (which is basically immobile) and the outflow tract.^1^ The inflow and outflow portions contract perpendicularly to each other: the proximal part longitudinally and the distal part circumferentially, in the orientation of their respective muscle fibres.^1^

Although the right ventricle can now be imaged and studied in several ways, two-dimensional (2D) guided M-mode echocardiography is an attractive tool due to its simplicity. Right ventricular (RV) long-axis movement represented by tricuspid annularplane systolic excursion (TAPSE) is an index that correlates excellently with RV ejection fraction obtained with radionuclide angiography and is independent of any geometric assumption.^2^ TAPSE provides a simple method for global RV functional assessment and is a strong predictor of prognosis in heart failure.^3^

For many years the right ventricle was grossly undervalued and considered to function mainly as a conduit, while its contractile performance was considered haemodynamically unimportant. Since the early 1950s, however, the relevance of the chamber in the maintenance of normal cardiac physiology was recognised in several cardiovascular disorders.^4^ Studies on RV function among patients with hypertensive heart disease (HHD) are rather few and have focused mainly on the diastolic function of the chamber.^5^,^6^ The primary aim of this study was to assess the systolic function of the right ventricle in patients with HHD, using TAPSE. A secondary aim was to compare the characteristics of patients found to have normal RV systolic function with those with abnormal function, as well as determine the correlates of TAPSE among the patients.

## Methods

The study was carried out in three echocardiography laboratories within the city of Kano in Nigeria: Aminu Kano Teaching Hospital (AKTH), Murtala Muhammad Specialist Hospital (MMSH) and a private centre (PC). Kano is a state in the north-western geopolitical zone of Nigeria and was previously reported to have the highest prevalence of systemic hypertension in the country.^7^

The research ethics committees of the study centres reviewed and approved the study protocol. All recruited patients gave informed written consent to participate in the study. The study conformed to the principles outlined in the Declaration of Helsinki, on the ethical principles for medical research involving human subjects.^8^

The study was cross sectional in design and patients were recruited serially from October 2008 to May 2009. After computing a minimum sample size using a validated formula,^9^ applying a prevalence of HHD in Kano of 56.7% (among patients referred for echocardiography),^10^ and a sample error of 10%, 186 patients were eventually recruited to improve the power of the study.

Information obtained from all recruited patients included relevant history, and findings from a physical examination, echocardiogram and electrocardiogram (ECG). Additional information obtained included recent (tested within the previous eight weeks) serum levels of creatinine and urea, and packed-cell volume (PCV) in venous blood.

Transthoracic echocardiography was performed using the Aloka cardiac ultrasound system (model SSD 4000 PHD) in AKTH, the Toshiba diagnostic ultrasound machine (model SSA 325A) in MMSH and the ATL Ultramark 9 ultrasound machine at the private centre, with 3.75-MHz sector transducers. The procedure was performed according to the recommendations of the American Society of Echocardiography,^11^,^12^ and by the same person (KMK) in all cases to avoid inter-observer variability. Patients were examined in the left lateral decubitus position. Right ventricular long-axis function (TAPSE) was recorded from the apical four-chamber view with the M-mode cursor positioned at the free wall angle of the tricuspid valve annulus. Right ventricular long-axis excursion amplitude (TAPSE) was taken from end-systole to end-diastole.^13^

Reduced TAPSE was defined as a value of < 15 mm, which has been found to be a strong predictor of death or emergency heart transplantation among heart failure patients.^3^ Normal RV systolic function was therefore defined as TAPSE of ≥ 15 mm (patients categorised as group 1), while values below 15 mm were considered abnormal (patients categorised as group 2). Evidence of raised pulmonary vascular resistance, suggestive of pulmonary hypertension, was defined as pulmonary valve (PV) acceleration time of < 100 ms.

Among the recruited hypertensive patients, a diagnosis of hypertensive heart disease was made if an abnormality was detected on the echocardiogram, which was causally related to hypertension and without an alternative explanation. These abnormalities included any of the three abnormal LV geometric patterns of HHD as defined by Ganau *et al.*,^14^ increased left atrial (LA) size and volume, and diastolic or systolic left ventricular dysfunctions. Systemic hypertension was defined according to the recommendation of the World Health Organisation/International Society of Hypertension (WHO/ISH), using the cut-off values of systolic/diastolic blood pressures (SBP/DBP) of ≥ 140/90 mmHg.^15^

Renal failure was simply defined as the presence of a serum creatinine concentration of ≥ 176 μmol/l (≥ 2 mg/dl).^15^ Anaemia was defined as packed-cell volume of < 39% in men and < 36% in women. History of tobacco smoking was considered a risk factor if smoking was daily, regardless of the dose. Excessive alcohol intake was defined as a weekly intake of more than 21 units for men and 14 units for women.^16^ The diagnosis of diabetes mellitus (DM) was based on WHO criteria.^17^ Cardiac rhythm disturbances were defined according to the recommendations of the American College of Cardiology/American Heart Association task force on clinical data standards.^18^ Ischaemic heart disease was excluded by the presence of all of the following: no history of angina, no ECG changes suggestive of myocardial infarction,^18^ and no regional wall motion abnormalities on echocardiography.

Data were analysed with SPSS version 11.5. Means and standard deviations were computed and presented for quantitative variables. The Student’s *t*-test, Wilcoxon rank-sum (z), Fisher’s exact and Chi-square (χ^2^) tests and measures of effect were used for comparison between groups as appropriate, with *p* < 0.05 regarded as significant. Pearson’s correlation (*r*) coefficient and the binary logistic regression model were used to analyse the associations between TAPSE and a number of variables.

## Results

A total of 186 patients were serially recruited and studied from the three centres over seven months (October 2008 to May 2009), comprising 89 males (47.85%) and 97 females (51.15%). The mean age of all patients was 55.94 ± 17.00 years.

Table 1 describes the baseline characteristics of the patients and compares patients with normal RV systolic function (group 1) with those with abnormal function (group 2). The table shows that compared with patients in group 2, group 1 patients were significantly younger and tended to have a shorter duration of hypertension. Group 1 patients also had a lower prevalence of features of heart failure (dyspnoea and peripheral oedema) and anaemia, and lower mean heart rate.

**Table 1.** Baseline Characteristics

	*Group 1 n = 131 (70.4%)*	*Group 2 n = 55 (29.6%)*	p*-value*	*All patients*
Males/females	67/64	22/33	0.199	89/97
Mean age (years)	53.50 ± 17.10	61.78 ± 15.38	0.002*	55.94 ± 17.00
Urban residence	112 (85.5%)	42 (76.4%)	0.141	154 (82.8%)
Duration of hypertension (years)	5.57 ± 6.62	7.87 ± 8.83	0.054	6.24 ± 7.21
Dyspnoea (NYHA III or IV)	46 (35.1%)	33 (60.0%)	0.002*	79 (42.5%)
Peripheral oedema	34 (26.0%)	30 (54.6%)	< 0.001*	64 (34.4%)
Smoking	6 (4.6%)	1 (1.8%)	0.676	7 (3.8%)
Alcohol	13 (9.9%)	4 (7.3%)	0.681	17 (9.1%)
Diabetes mellitus	11 (8.4%)	5 (9.1%)	0.878	16 (8.6%)
Stroke/TIA	15 (11.5%)	2 (3.6%)	0.103	17 (9.1%)
Anaemia	19 (14.5%)	18 (32.7%)	0.016*	37 (19.1%)
Renal failure	20 (15.3%)	15 (27.3%)	0.101	35 (18.8%)
Systolic BP (mmHg)	156.61 ± 30.50	149.25 ± 28.16	0.150	154.54 ± 29.97
Diastolic BP (mmHg)	95.11 ± 20.26	93.19 ± 18.51	0.570	100.88 ± 86.80
Pulse pressure (mmHg)	61.54 ± 22.95	56.06 ± 18.35	0.141	60.00 ± 21.85
Heart rate	88.52 ± 16.45	102.58 ± 19.45	< 0.001*	92.47 ± 18.41
PCV (%)	28.67 ± 10.67	30.46 ± 8.61	0.534	29.57 ± 9.63
Serum urea (mmol/l)	14.30 ± 11.21	14.16 ± 10.98	0.963	14.24 ± 11.00
Serum creatinine (μmol/l)	585.72 ± 789.49	333.33 ± 383.36	0.155	477.55 ± 654.72

*p-value statistically significant; PCV: packed-cell volume. All values are expressed as means ± standard deviations, or as proportions, or as numbers with percentages in parentheses.

The mean TAPSE in all patients was 18.30 ± 5.82 mm. Table 2 compares the echocardiographic features of the two groups. Patients in group 2 had a significantly larger right ventricle, left ventricle and left atrium, and lower mean TAPSE. Indices of LV long-axis function (LV lateral and septal APSE) were also significantly lower among group 2 patients.

**Table 2.** Echocardiographic Features

	*Group 1 n = 131 (70.4%)*	*Group 2 n = 55 (29.6%)*	p*-value*
RVOTd (mm)	28.83 ± 5.50	31.02 ± 7.03	0.024*
TAPSE (mm)	21.00 ± 4.60	11.82 ± 2.24	< 0.001*
Left atrium (mm)	38.31 ± 7.50	43.04 ± 7.76	< 0.001*
IVSd (mm)	11.25 ± 3.85	10.65 ± 4.43	0.358
LVPWd (mm)	9.64 ± 3.02	9.22 ± 2.99	0.383
LVEDD (mm)	51.69 ± 11.45	57.07 ± 13.51	0.006*
LVEF (%)	57.88 ± 16.82	42.42 ± 19.00	< 0.001*
PV acceleration time (ms)	119.71 ± 39.54	86.41 ± 27.10	< 0.001*
LV lateral APSE (mm)	12.38 ± 4.11	8.80 ± 2.52	< 0.001*
Septal APSE (mm)	11.50 ± 3.99	7.06 ± 4.04	< 0.001*

*p-value statistically significant; LVEF: left ventricular ejection fraction; RVOTd: right ventricular out-flow tract dimension at end-diastole; TAPSE: tricuspid annular plane systolic excursion; IVSd: interventricular septal thickness at end-diastole; LVPWd: left ventricular posterior wall thickness at end-diastole; LVEDD: left ventricular end-diastolic dimension; PV: pulmonary valve. All values are expressed as means ± standard deviations.

Table 3 shows the findings in the resting ECG of the patients. Atrial arrhythmias were more prevalent among group 2 patients, and the most frequent were atrial fibrillation or flutter. Group 2 patients also had statistically significant shorter PR and longer QT_c_ intervals.

**Table 3.** Findings In The Resting Electrocardiograms

*Characteristic*	*Group 1 n = 30 (16.1%)*	*Group 2 n = 156 (83.9%)*	p*-value*
PR interval (ms)	165.09 ± 30.96	136.15 ± 42.41	< 0.001*
QRS duration (ms)	94.91 ± 18.99	99.84 ± 23.59	0.218
QT_c_ interval (ms)	432.17 ± 47.31	455.97 ± 42.71	0.009*
Atrial fibrillation/ flutter	4 (3.1%)	11 (20.0%)	< 0.001*
Other SVT	1 (0.8%)	3 (5.5%)	0.043*
Complete heart block	2 (1.5%)	0	–
Complete LBBB	4 (3.1%)	4 (7.3%)	0.196
Complete RBBB	4 (3.1%)	1 (1.8%)	0.635
1st degree heart block	8 (6.1%)	1 (1.8%)	0.214

SVT: supraventricular tachycardias; LBBB and RBBB: left and right bundle branch block respectively. All values are expressed as means ± standard deviations or as numbers with percentages in parentheses.

Table 4 describes the correlates of TAPSE. The strongest correlates of TAPSE were its corresponding indices of long-axis function of the left ventricle [lateral and septal annular-plane systolic excursion (APSE)].

**Table 4.** Correlates Of Tapse

	*TAPSE (mm)*	
*Variable*	*r*	p-value*
Age (years)	+0.385	< 0.001
Duration of hypertension (years)	–0.202	0.007
Heart rate	–0.263	0.001
PR interval (ms)	+0.216	0.025
QT_c_ interval (ms)	–0.256	0.004
RVOTd (mm)	–0.223	0.002
Left atrium (mm)	–0.232	0.001
LVEDD (mm)	–0.207	0.004
LVESD (mm)	–0.354	< 0.001
LVEF (%)	+0.462	< 0.001
PV acceleration time (ms)	+0.440	< 0.001
LV lateral APSE (mm)	+0.534	< 0.001
Septal APSE (mm)	+0.541	< 0.001

*p-value statistically significant; LVEF: left ventricular ejection fraction; RVOTd: right ventricular out-flow tract dimension at enddiastole; TAPSE: tricuspid annular plane systolic excursion; LVEDD: left ventricular end-diastolic dimension; PV: pulmonary valve.

In the logistic regression model controlling for other confounding factors, independent predictors of reduced TAPSE were age [odds ratio (OR) = 1.035; confidence interval (CI) = 1.012–1.058 years; *p* = 0.002], peripheral oedema (OR = 2.921; CI = 1.036–8.239; *p* = 0.043), LA diameter (OR = 1.061; CI = 1.001–1.125 mm; *p* = 0.046), LV end-diastolic diameter (LVEDD) (OR = 0.882; CI = 0.811–0.959 mm; *p* = 0.003), LV end-systolic diameter (LVESD) (OR = 1.133; CI = 1.058–1.214 mm; *p* < 0.001), LV out-flow tract (LVOT) diameter (OR = 0.855; CI = 0.754–0.970 mm; *p* = 0.015), and septal APSE (OR = 0.777; CI = 0.641–0.943 mm; *p* = 0.011).

## Discussion

Although systemic hypertension is one of the most researched subjects in medicine, the literature on right ventricular systolic function among hypertensives is quite scanty,^6^ especially among Africans. This study showed that almost one-third of patients with HHD (29.6%) on echocardiography in Kano had RV systolic dysfunction in the form of reduced RV long-axis excursion (TAPSE).

In a sample population of patients with heart failure of various aetiologies, including systemic hypertension, Puwanant *et al*. recently reported that 58% of the patients had reduced TAPSE (< 15 mm), in contrast to our finding of 29.6%.^19^ However, our study was exclusively on patients with HHD, and a history of diabetes mellitus (DM) was found in only 8.6%. In comparison, 51% of the patients in the series by Puwanant *et al*. had coronary artery disease, 37% had DM and 32.5% had cardiomyopathies. In addition, the patients in the latter study were older than ours (mean age of 72 ± 14 vs 55.94 ± 17.00 years), and we have shown that older age predicted reduced TAPSE. Furthermore, differences in the aetiology of heart disease in the two studies could have amplified the disparities in TAPSE.^19^

It is possible that RV disease develops in parallel with a similar process on the left side in hypertensive patients, likely as a result of ventricular interdependence. This is supported by the close correlation we found between the TAPSE and the indices of LV long-axis function, lateral and septal APSE (*r* = +0.534 and +0.541, respectively, *p* < 0.001 for both). Ventricular interdependence is defined as the forces that are transmitted directly from one ventricle to the other through the myocardium and pericardium, independent of neural, humoral or circulatory effects. It is a consequence of the close anatomical association between the ventricles, which are encircled by common muscle fibres, they share a septal wall, and are enclosed within the pericardium.^20^ Future studies are however needed to corroborate our finding and to explore other explanations.

To the best of our knowledge, our study is the first, at least in Africans, to group and compare hypertensives based on normal or reduced TAPSE. We have shown that patients in group 2 with reduced TAPSE were older (*p* = 0.002), had longer duration of hypertension (*p* = 0.054) and higher prevalence rates of other indices associated with a poor prognosis. These indices include higher prevalence rates of moderate to severe dyspnoea (*p* = 0.002), peripheral oedema (*p* < 0.001) and anaemia (0.016). In addition, group 2 patients also had a shorter PR interval (*p* < 0.001), a longer QT_c_ interval (*p* = 0.009), and a higher prevalence of supraventricular tachycardias (*p* = 0.043), including atrial fibrillation and flutter (*p* < 0.001).

Previous studies have shown that atrial tachyarrhythmias are the most common arrhythmias encountered in patients with RV failure. In the setting of acute RV failure or severe RV dysfunction, atrial tachyarrhythmias often lead to haemodynamic instability. Other studies have demonstrated that atrial flutter or atrial fibrillation is associated with an increased risk of morbidity or mortality in patients with RV myocardial infarction, pulmonary hypertension, and congenital heart disease (CHD). Right atrial dilatation and remodelling and postsurgical scars within the atria, as in postoperative CHD, represent important substrates for atrial flutter.^4^,^21^-^24^ Our study has demonstrated the propensity of HHD patients with RV systolic dysfunction to arrhythmias, but a prospective study with a larger sample size would be needed to corroborate the finding.

The strongest correlate to TAPSE was the septal mitral annular-plane systolic excursion (*r* = –0.541; *p* < 0.001). Other workers have found a strong correlation between TAPSE and RV ejection fraction,^2^ as well as indices of RV diastolic function.^25^

In the logistic regression model, age was among the predictors of TAPSE (OR = 1.035; *p* = 0.002), reconfirming the importance of age in predicting cardiovascular morbidity and mortality among persons with high blood pressure.^15^ Peripheral oedema is another clinical variable that was found to predict reduced TAPSE (OR = 2.921; *p* = 0.043), and its presence suggests the presence of RV failure. This finding is important, given that peripheral oedema is an easy-to-identify physical sign, and its presence increased the odds of reduced TAPSE almost three-fold.

The limitations of our study include the small sample size of patients with RV systolic dysfunction, as well as the limitation that is inherent to the study, which is the use of M-mode and 2D echocardiography to study the RV. Studies have shown the superiority of magnetic resonance imaging over other techniques for studying the right ventricle.^26^ However, echocardiography still has acceptable sensitivity, is widely available and affordable, and therefore has an important role in studying the right ventricle despite its limitation. In addition, TAPSE is easy to obtain, is reproducible, and is without significant inter-observer variability.^2^ To minimise this, all our echocardiograms were performed by a single individual.

## Conclusion

This study found that 29.6% of patients with HHD in Kano had RV systolic dysfunction in the form of reduced tricuspid annularplane systolic excursion. Such patients tended to be older, had evidence of worse LV systolic function and a higher prevalence of supraventricular arrhythmias. Several variables were found to correlate significantly with TAPSE, but the association was strongest with septal mitral annular-plane systolic excursion. Predictors of reduced TAPSE included age, peripheral oedema and several echocardiographic variables. The presence of peripheral oedema increased the odds of reduced TAPSE about three-fold. We recommend that assessment of RV systolic function should be carried out routinely in all hypertensive patients requiring echocardiographic evaluation.
